# A Rare Cause of Recurrent Vaginal Hydrocele: Herniating Mesenteric Hydatid Cyst

**Published:** 2017

**Authors:** Yosra KERKENI, Sondes SAHLI, Manef GASMI, Nada SGHAIROUN, Mourad HAMZAOUI

**Affiliations:** Dept. of Pediatric Surgery A, Children’s Hospital Bechir Hamza, Faculty of Medicine of Tunis, University of Tunis El Manar, Tunis, Tunisia

**Keywords:** Hydatid disease, Cyst, Mesenterium, Migration, Herniation, Tunisia

## Abstract

Echinococcosis is a multisystem disease and has propensity to involve any organ, an unusual anatomical site, and can mimic any disease process. The hydatid cyst of the mesenteries known to occur secondary to hepatic involvement but occasional cases of his primitive form has also been reported. We report here one such case of primitive mesenteric hydatid cyst herniated through inguinal canal in a 5-yr-old boy, admitted to our Pediatric Surgery Department of Children’s Hospital in Tunis, Tunisia in 2015.

## Introduction

Hydatid disease (HD) is a parasitic infection caused by the larvae of *Echinococcus granulosus*. It affects almost every region of the body. In children, the lungs are the most common site (64%) of hydatid cyst followed by liver (28%) (1). The hydatid cyst of the mesentery is a rare localization of HD; most often, it is secondary to a hydatid cyst of the liver. His primitive form is exceptional and would be due to hematogenous spread by arterial way (2). Although very unusual locations are described, this is the first report of mesenteric hydatid cyst herniated through inguinal canal in children.

## Case report

A 5-yr-old boy was admitted to the Pediatric Surgery Department of our Children’s Hospital in Tunis on Sep 2015. Informed consent and University approval were taken.

**Fig. 1:**
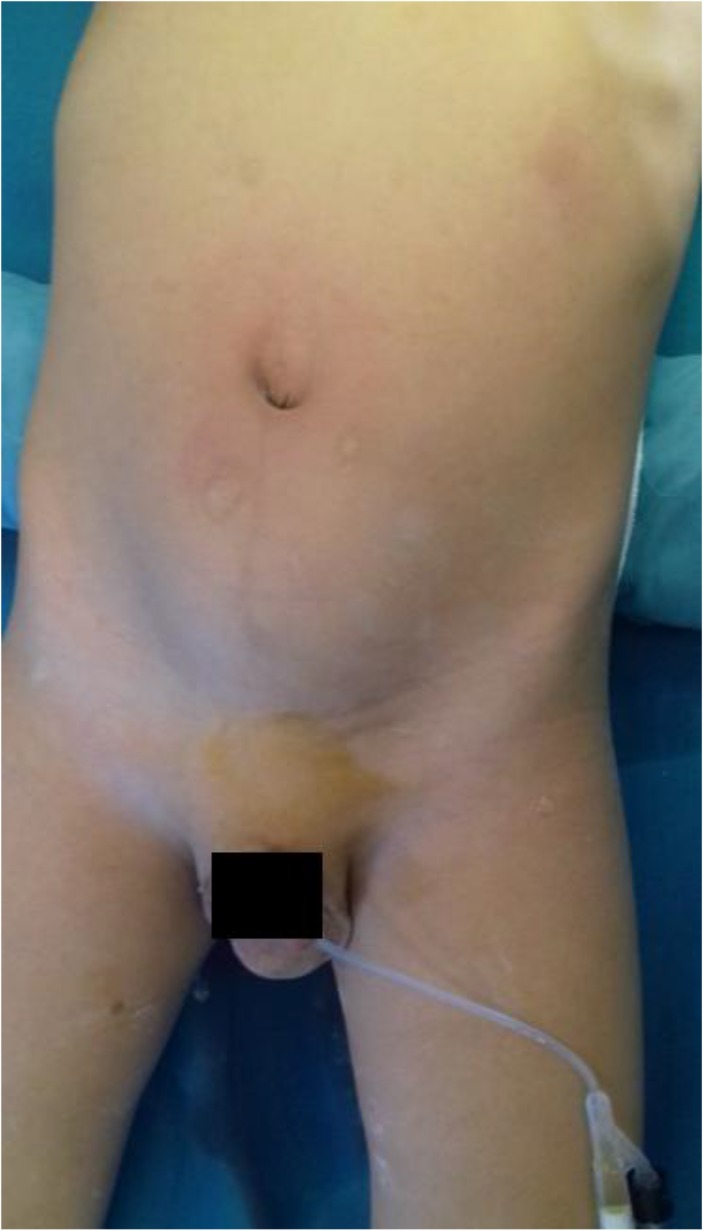
Hypogastric mass associated to a communicating left vaginal hydrocele

**Fig. 2:**
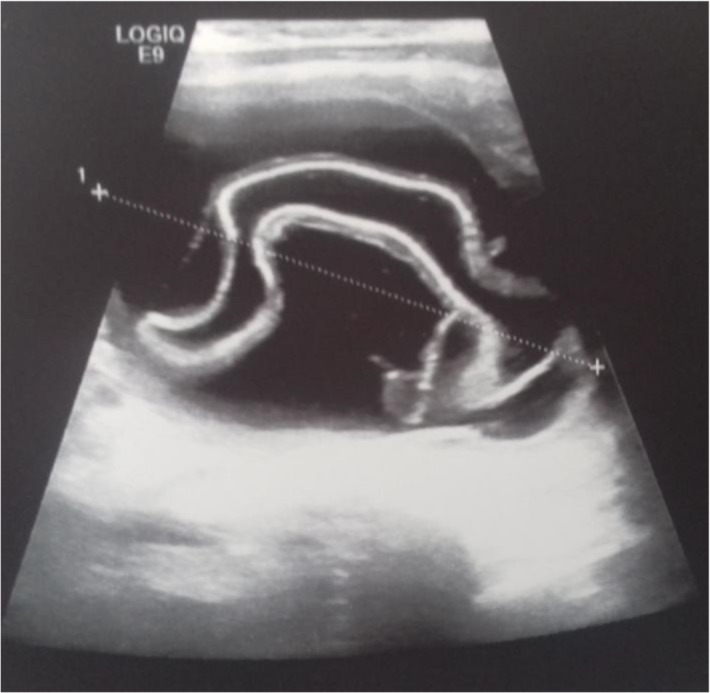
Abdominal ultrasound showed a well-defined, anechoic cystic mass, seat of a floating membrane

**Fig. 3:**
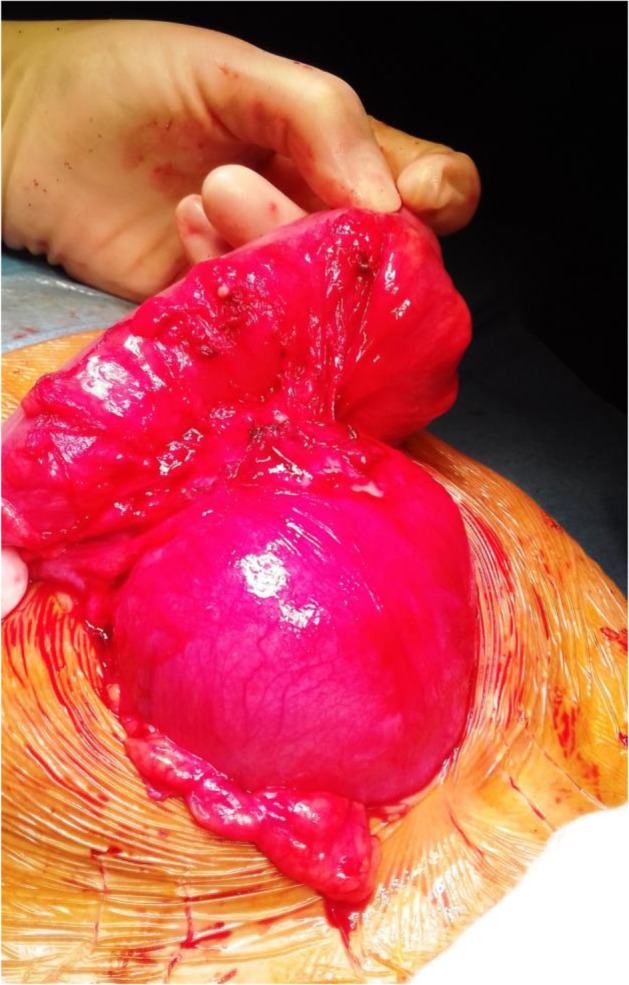
Large cystic mass arising from sigmoid mesocolon

He presented to the clinic with abdominal mass. He had a history of left recurrent hydrocele operated on twice. On examination, a mass approximately 6 × 8, 5 cm in diameter was identified in hypogastric region. External genitalia examination was suggestive of recurrent communicating left vaginal hydrocele (Fig. 1). Abdominal ultrasound showed a well-defined, anechoic cystic mass, seat of a floating membrane (Fig. 2). An intraperitoneal hydatid cyst, located above and behind the bladder and a complete persistent peritonealvaginal duct were concluded. Chest radiography and ultrasonography showed no coexisting thoracic or liver hydatid disease. Laparotomy was performed, and upon entering the peritoneal cavity, we found a large cystic mass arising from sigmoid mesocolon (Fig. 3).

Its anterior wall filled the left inguinal ring. As for liver hydatid cyst, we performed PAIR (Puncture, aspiration, injection, and respiration) technique. Saline pads were packed beneath the cyst, elevating it to a fixed position, and the intestines were protected with these pads. Hydatid fluid and protoscolices were aspirated with needle aspirator. A 20% hypertonic saline solution was used to deactivate the cyst content. The solution was left inside the cyst about 10 min to kill the protoscolices. After evacuating the cystic contents, cystotomy was performed.

The typical germinative membrane of a hydatid cyst was identified and removed. The cavity was explored, revealing a communication with internal inguinal ring. The inguinal approach showed the extension of the pericystic layer through inguinal canal. A very careful dissection of the structures of the cord was performed to isolate the processus. The communication was ligated at the internal ring. After dissection of the inguinal part of the cyst, total excision was performed. Given the risk of injuring major vessels passing just outside the posterior pericystic layer of the mesenteric cyst, we opted for a partial cystectomy. A drain was then placed into the residual cavity. The examination of the fluid aspirated from the cyst demonstrated free protoscolices showed hooklets. On histopathological examinations, the cyst wall had a laminated membrane with inner germinal layer containing degenerated eosinophilic protoplasmic mass. The inguinal part of the cyst revealed similar histopathological findings of the hydatid cyst. The postoperative course was uneventful and the child had a full recovery.

The patient received albendazole, 10 mg kg−1 d−1 for 3 months postoperatively. No recurrence was encountered during the 8-month follow-up period.

## Discussion

Hydatid cyst is a parasitic disease that caused by the larval stage of *Echinococcus granulosus*. It is still a considerable health problem in the world (1, 2). Hydatid cysts can be located in any organ or tissue in the body, such as the posterior mediastinum, intestinal mesentery, parotid gland, thigh, retroperitoneal space, adrenal gland, salivary glands, scrotum, and spermatic cord (3). Therefore, when dealing with intraperitoneal cysts, hydatid cyst should be suspected. The hydatid cyst of the mesentery is a rare localization of HD; most often, it is secondary to a hydatid cyst of the liver. His primitive form was exceptional and would be due to hematogenous spread by arterial way (2). Complications of hydatid cyst include rupture and superinfection of type I and II cysts (4). Rupture, that can be contained, communicating or direct, happens in 50 to 90% of cases (4). In contained rupture, the endocyst ruptures and becomes detached from the pericyst. The opening of hydatid cysts into an anatomical diversion structure defines a communicating rupture (4, 5). The present report described a rare complication consisting in the herniation of mesenteric hydatic cyst through inguinal canal. In some patients, due to gravity and difference of pressure, active hydatid cysts may migrate and herniate through anatomical cavities (5, 6).

Inguinal herniation of mesenteric hydatid cyst has not been reported yet, however, it should be considered especially in countries where echinococcosis is endemic, such as South East Asia, Mediterranean countries, the Middle East, and the Far East (1).

There are no specific signs or symptoms attributed to hydatid disease with uncommon localization (3). Because the growth of the cyst is slow, intraperitoneal cystic mass can go unnoticed. However, careful examination for intraabdominal disease could avoid an erroneous diagnosis of a simple recurrent vaginal hydrocele. The possibility of herniation in patients with HD should be considered because arriving at a correct preoperative diagnosis is important for the surgeon so that he/she can avoid the possibility of an inadvertent spillage of the parasite during the operation. Total cyst excision is the recommended operation of choice, but if vital or important structures are in close contact with the cyst (mesentery vessels, in our case); partial excision can also be done (1–3). The use of hypertonic saline into the hydatid cyst has been considered a safe and effective form of scolicidal agent but it may result in major complications such as hypernatremia (1). Postoperative chemotherapy is generally advised for complete cure (1).

Only two cases with herniation of intraperitoneal hydatid cyst by inguinal canal have been reported in English literature and they were one case of primary hydatid cysts and another case of secondary hydatid cysts of the female genitaliain adults (6). This case is the first report that presents a mesenteric hydatid cyst herniating through inguinal canal in children.

## Conclusion

HD should be remembered in the differential diagnosis of cystic pelvic masses whatever the age of the patient. Recurrent vaginal hydrocele, when it is combined with an intraabdominal palpable mass, should be a reminder of HD in endemic regions.
